# Novel Small Molecule Agonist of TGR5 Possesses Anti-Diabetic Effects but Causes Gallbladder Filling in Mice

**DOI:** 10.1371/journal.pone.0136873

**Published:** 2015-08-27

**Authors:** Daniel A. Briere, Xiaoping Ruan, Christine C. Cheng, Angela M. Siesky, Thomas E. Fitch, Carmen Dominguez, Sonia Gutierrez Sanfeliciano, Carlos Montero, Chen S. Suen, Yanping Xu, Tamer Coskun, M. Dodson Michael

**Affiliations:** 1 Lilly Research Laboratories, Eli Lilly and Co., Indianapolis, Indiana, United States of America; 2 Centro de Investigación, Eli Lilly and Company, Alcobendas, Spain; Beckman Research Institute of City of Hope, UNITED STATES

## Abstract

Activation of TGR5 via bile acids or bile acid analogs leads to the release of glucagon-like peptide-1 (GLP-1) from intestine, increases energy expenditure in brown adipose tissue, and increases gallbladder filling with bile. Here, we present compound 18, a non-bile acid agonist of TGR5 that demonstrates robust GLP-1 secretion in a mouse enteroendocrine cell line yet weak GLP-1 secretion in a human enteroendocrine cell line. Acute administration of compound 18 to mice increased GLP-1 and peptide YY (PYY) secretion, leading to a lowering of the glucose excursion in an oral glucose tolerance test (OGTT), while chronic administration led to weight loss. In addition, compound 18 showed a dose-dependent increase in gallbladder filling. Lastly, compound 18 failed to show similar pharmacological effects on GLP-1, PYY, and gallbladder filling in *Tgr5* knockout mice. Together, these results demonstrate that compound 18 is a mouse-selective TGR5 agonist that induces GLP-1 and PYY secretion, and lowers the glucose excursion in an OGTT, but only at doses that simultaneously induce gallbladder filling. Overall, these data highlight the benefits and potential risks of using TGR5 agonists to treat diabetes and metabolic diseases.

## Introduction

In 1902, Bayliss and Starling speculated that an endocrine substance arising from the gut after ingestion of nutrients induces secretions from the pancreas [1]. Today, the “incretin effect” describes the phenomenon where insulin secretion is profoundly more robust following glucose ingestion compared to the insulinotropic response achieved by parenteral administration of intravenously infused glucose [2,3]. In fact, it is estimated that 50% to 70% of the secretion of insulin from the pancreas following a meal occurs due to incretin action [3]. Two hormones with known incretin activity are glucagon-like peptide-1 (GLP-1) released from intestinal L-cells and glucose-dependent insulinotropic peptide (GIP) released from intestinal K-cells [4]. However, once secreted in response to a meal, circulating levels of intact GLP-1 and GIP decrease rapidly due mainly to cleavage by dipeptidyl peptidase-4 (DPP4) [4]. In addition to GLP-1 and GIP, peptide YY (PYY) is released from intestinal L-cells upon nutrient stimulation, where it exhibits effects by reducing gastric motility and emptying, reducing appetite, and increasing insulin sensitivity [5,6]. Further, GLP-1 and PYY have been shown to act synergistically to slow gastric emptying and inhibit food intake [7,8].

Luminal nutrients act through a variety of mechanisms to stimulate incretin and PYY secretion from enteroendocrine cells, including sodium/glucose co-transporter-1 (SGLT-1) for glucose, as well as numerous G-protein coupled receptors (GPCR), such as G_q_-coupled receptors (e.g. GPR40, GPR41, GPR43, and GPR120) for fatty acids and G_s_-coupled receptors (e.g. GPR119) for fatty acid derivatives [9]. In addition to their stimulation of hormone secretion, lipids in the diet also cause the release of bile acids from the gallbladder that help facilitate processing of dietary fat and fat soluble vitamins [10]. In humans, bile acids primarily include cholic acid and chenodeoxycholic acid, with lithocholic acid and deoxycholic acid being secondary; mice have similar primary bile acids but convert chenodeoxycholic acid to muricholic acid [11]. After secretion, approximately 95% of bile acids are reabsorbed and transported back to the liver via a process known as “enterohepatic circulation” [12].

Recently, new insights into the biological activity of bile acids have been revealed, including the discovery of a G_s_-protein coupled receptor designated TGR5 [13,14]. TGR5 (also known as GPBAR1 and GPR131) is a GPCR that is activated by bile acids and expressed in intestine, brown adipose tissue (BAT), and gallbladder [13–15]. Initial work using bile acids and the enteroendocrine GLP-1 secreting cell lines GLUTag, STC-1, and NCI-H716 demonstrated a role of TGR5 activation in the stimulation of cAMP and release of GLP-1 and PYY [16–19]. Subsequently, bile acids were shown to increase energy expenditure in mice [20]. As a result of these efforts, TGR5 agonists have been proposed as novel treatments for obesity and type 2 diabetes.

Early efforts aiming at identifying TGR5 agonists focused on bile acids and bile acid analogs, including INT-777 (6α-ethyl-23(S)-methylcholic acid, S-EMCA, MW 450.65), a semisynthetic derivative of cholic acid first described in 2009 [21]. Thomas et al. showed that INT-777 increases GLP-1 secretion from L-cells, may possess some effects on acute GLP-1 secretion *in vivo*, leads to modest weight loss with chronic administration, and has the potential for improvement in lowering glucose excursion in an OGTT in chronically treated mice [22]. Overall, effects observed with administration of bile acids and bile acid analogs, such as INT-777, support TGR5 agonism as a treatment for metabolic disease.

TGR5 is expressed in gallbladder at levels higher than any other tissue, appears localized to the gallbladder epithelium, and is involved in chloride and fluid secretion [23]. Moreover, *Tgr5* knockout mice (KO) have been shown to be resistant to gallstone formation induced from a lithogenic diet [15]. Together, these data urged an investigation of TGR5 agonism on gallbladder physiology. In 2011, Li et al. reported that two bile acids, cholic acid and lithocholic acid, as well as the bile acid derivative INT-777, caused smooth muscle relaxation and stimulated gallbladder filling with bile [24]. Together, these results suggest that while a TGR5 agonist may stimulate GLP-1 secretion and subsequent improved glucose homeostasis, there may be potential to exacerbate gallstone formation or other gallbladder-related conditions.

In this study, we utilized a novel TGR5 agonist (compound 18) as well as *Tgr5* knockout (KO) mice to investigate the potential benefits as well as risks of TGR5 agonism. Our results demonstrate that compound 18 induces GLP-1 and PYY secretion, promotes weight loss, and lowers glucose in OGTT experiments, but only at doses that also cause gallbladder filling. Moreover, by employing *Tgr5* KO mice, we show the effects of compound 18 on GLP-1 and PYY secretion and gallbladder filling are TGR5-dependent. Overall, these data suggest that the glucoregulatory benefits for TGR5 agonists in rodents for the treatment of diabetes and metabolic syndrome likely cannot be separated from concurrent pharmacology in the gallbladder.

## Materials and Methods

### RT-PCR

Total RNA was isolated from mouse tissues, STC-1 and NCI-H716 cells using RNeasy 96 kit (QIAGEN) and reverse transcribed into cDNA using High-Capacity Reverse Transcription Kit (Applied Biosystems). Human tissue cDNAs were from Clontech Laboratories, Inc. and human gallbladder cDNA was from Invitrogen. Both mouse *Gpbar1* (gene encoding TGR5) primer/probe (Applied Biosystems: Mm00558112_s1), human *Gpbar1* primer/probe (Applied Biosystems: Hs00544894_m1), mouse *Gcg* (transcript which gives rise to GLP-1, Applied Biosystems: Mm00801712_m1), and TaqMan Universal PCR Master Mix (Applied Biosystems) were used for TaqMan gene expression assay of reverse transcribed samples and analyzed by SDS 2.2. β-actin was used as an internal control.

### Cell culture

HEK-293 cells were obtained from ATCC and a clonal HEK-293 line overexpressing mouse TGR5 was prepared. Both were maintained in Dulbecco’s modified Eagle’s medium (DMEM) containing 10% fetal bovine serum (FBS) and penicillin /streptomycin. Mouse STC-1 cells were obtained from ATCC under agreement with the Cold Spring Harbor Laboratories [25] and maintained in DMEM containing 10% FBS and penicillin /streptomycin. NCI-H716 cells were obtained from ATCC and were maintained in RPMI-1640 with 10% FBS. NCI-H716 cells were cultured in 96 well plate coated with Matrigel (BD). After 72 hours, cAMP production stimulated with TGR5 agonists was measured using HTRF cAMP assay kit (CISBIO). The level of cAMP in suspensions of STC-1 cells stimulated with increasing concentrations of TGR5 agonists was also determined. After 2 day-culturing, GLP-1 secretion in Matrigel-differentiated NCI-H716 or STC-1 cells was induced by increasing concentrations of TGR5 agonists. The production of GLP-1 was determined using a GLP-1 Assay (7–36) amide kit (Mesoscale Discovery) following the manufacturer’s instructions. For both cAMP and GLP-1, the maximal response for each compound was set to 100% in each cell line.

### Ethics statement

Animals were studied and maintained in accordance with the Institutional Animal Care and Use Committee (IACUC) of Eli Lilly and Company, and the Guide for the Use and Care of Laboratory Animals by the National Institutes of Health. All animal studies described herein were approved by the IACUC of Eli Lilly and Company.

### Animal husbandry

Mice were singly housed in microisolator cages on wood chip bedding with standard food (5008 Teklad Global Diet, Harlan, Indianapolis) or high-fat food (diet-induced obese mice only; Teklad TD95217, Harlan, Indianapolis), and deionized water available *ad libitum*. Lights were on a “normal” 12:12 hour (6 AM to 6 PM on) light: dark cycle, or “reversed” 12:12 hour (9 AM to 9 PM off) light: dark cycle, and temperature and relative humidity were maintained between 21 and 23°C and 45 and 65%, respectively. Male C57BL/6 mice at 8–10 weeks of age were purchased from Harlan Sprague-Dawley (Indianapolis, IN). *Tgr5* KO mice and their wild-type littermates were obtained from a private breeding colony from Taconic at 8–10 weeks of age. Diet-induced obese (DIO) mice were ordered from Harlan Sprague-Dawley at 20–24 weeks of age.

### Compound preparation

INT-777 and sitagliptin were synthesized at Eli Lilly and Company using traditional medicinal chemistry methods and were formulated in 20% Captisol w/v in water. Compound 18 was identified through an internal effort, synthesized using traditional medicinal chemistry methods, and formulated as a solution in 20% Captisol w/v in water with the addition of up to 2 molar equivalents of HCl to make the salt. All compounds were prepared the evening before the experiment and stored at 4°C.

### Acute in vivo incretin secretion assays

Overnight-fasted C57BL/6 mice were orally dosed with compounds, followed by euthanasia by CO_2_ asphyxiation and cardiac puncture to collect blood into pre-chilled EDTA plasma tubes containing aprotinin and a DPP4 inhibitor at 0.25, 0.5, 1.5, and 3 hours post dose. Plasma was separated by centrifugation, aliquoted, and analyzed for GLP-1 (X-36) amide (Mesoscale Discovery) and total PYY (Millipore). Resulting raw data was used to generate area under the curve analyses (AUCs). Quantification of INT-777 or compound 18 concentrations in plasma was determined using LC-MS/MS methods on a Sciex API4000 instrument at Quintiles (Plainfield, IN).

### Oral glucose tolerance tests

For measurement of glucose only, overnight-fasted C57BL/6 mice in a normal light cycle were orally dosed with vehicle or compound, followed by an oral bolus of 3 g/kg glucose at 15 minutes post compound. At 0, 20, 40, 60, and 120 minutes post glucose dose, blood glucose was assessed via glucometers. Resulting raw data was used to generate AUCs. For measurement of hormones and glucose, overnight-fasted C57BL/6 mice acclimated for two weeks to a reverse light cycle were orally dosed with vehicle or compound, followed by an oral bolus of 3 g/kg glucose at 15 minutes post compound. At 0, 2, 5, 10, 20, 40, and 60 minutes post glucose dose, blood glucose was assessed via glucometers, followed immediately by collection of plasma for active GLP-1 (Mesoscale Discovery), insulin (Mesoscale Discovery), and total PYY. Glucose tolerance tests in diet-induced obese (DIO) C57BL/6 mice were performed in overnight-fasted animals dosed either acutely or after 2-week administration with vehicle or compound 18 at 60 mg/kg, followed 30 minutes post compound by an oral bolus of 2 g/kg glucose. At 0, 15, 30, 60, and 120 minutes post glucose dose, blood glucose was assessed via glucometers. Resulting raw data was used to generate AUCs.

### Body composition

Body composition of mice was determined using Quantitative Nuclear Magnetic Resonance analysis (ECHO MRI, 3–1 Composition Analyzer; Echo Medical Systems, Houston, TX). DIO mice were randomized by body weight and fat mass prior to administration of compounds. Changes in body weight, cumulative food intake, and fat and lean mass were recorded for up to a two week period.

### Three-day studies on GLP-1 and gallbladders

C57BL/6 mice, *Tgr5* KO mice, or their wild-type littermate controls were orally dosed with vehicle or compounds twice per day for 3 days, with overnight fasting performed on the evening of day 2. On the morning of day 3, animals were dosed orally with compounds and then euthanized by CO_2_ asphyxiation at 30 minutes after the fifth dose. Plasma was collected for analysis of GLP-1 (X-36) amide as mentioned above, followed immediately by incision into the abdominal cavity to remove and weigh bile from the gallbladder.

### Statistical analysis

Data are represented as the mean ± SEM and were compared using JMP9 using Dunnett’s method (versus vehicle), Tukey-Kramer, or t-test. The null hypothesis was rejected at p < 0.05.

## Results

### TGR5 gene is expressed in human and mouse enteroendocrine tissues and cell lines

**Fig 1:**
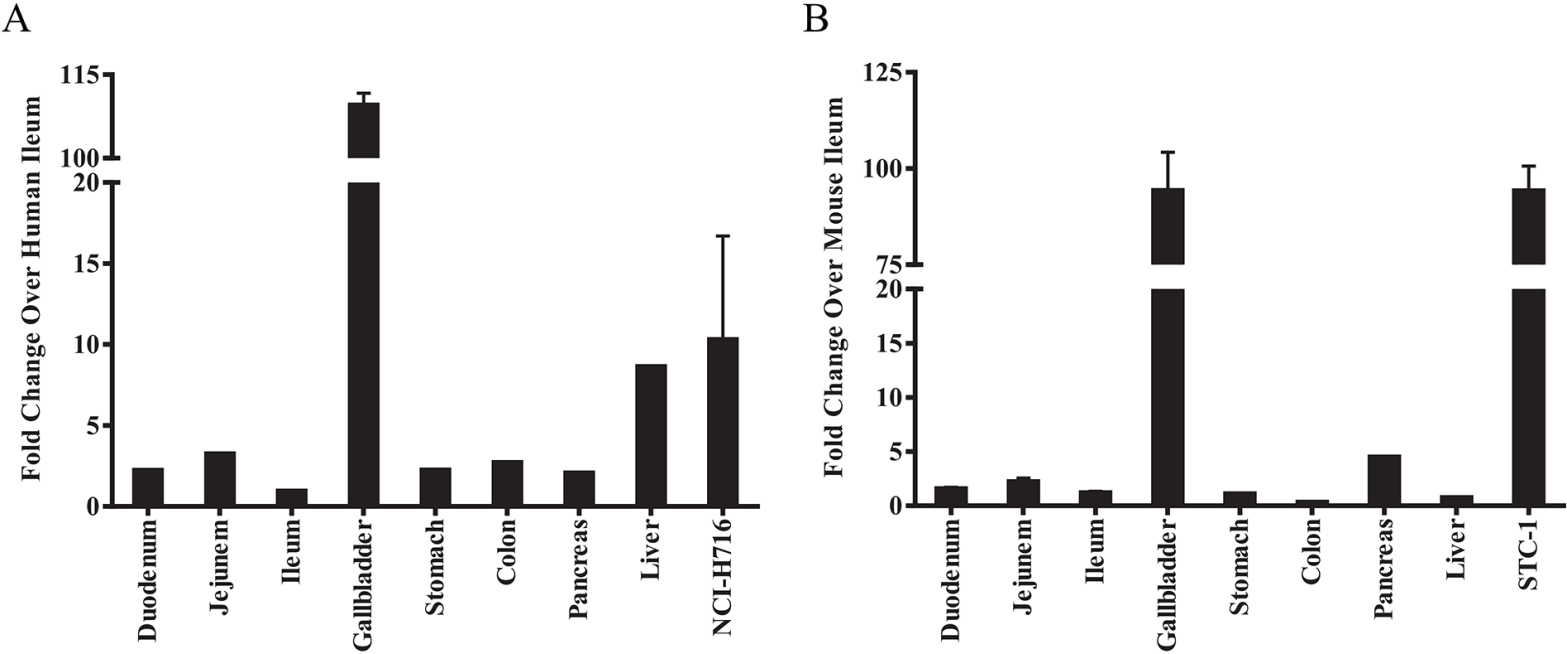
Expression of TGR5 in human and mouse tissues and enteroendocrine cell lines NCI-H715 and STC-1. TaqMan gene expression assay using total RNA isolated from human (A) and mouse (B) tissues as well as human NCI-H716 (A) and mouse STC-1 cells (B). β-actin was used as an internal control. Data represent the fold over ileum for each species independently, since different probe/primer sets were used. Values represent the mean +/- SEM.

RT-PCR was performed to confirm gene expression profiles and tissue distribution of the *TGR5* gene in human and mouse tissues, as well as two enteroendocrine cell lines. TGR5 mRNA was detected in all tissues and cell samples examined, with highest levels observed in gallbladder, intestinal tissues, and liver (Fig 1). In addition, TGR5 was also highly expressed in the human enteroendocrine cell line NCI-H716 (Fig 1A) and mouse cell line STC-1 (Fig 1B).

### Compound 18 is a potent and selective TGR5 agonist

**Fig 2:**
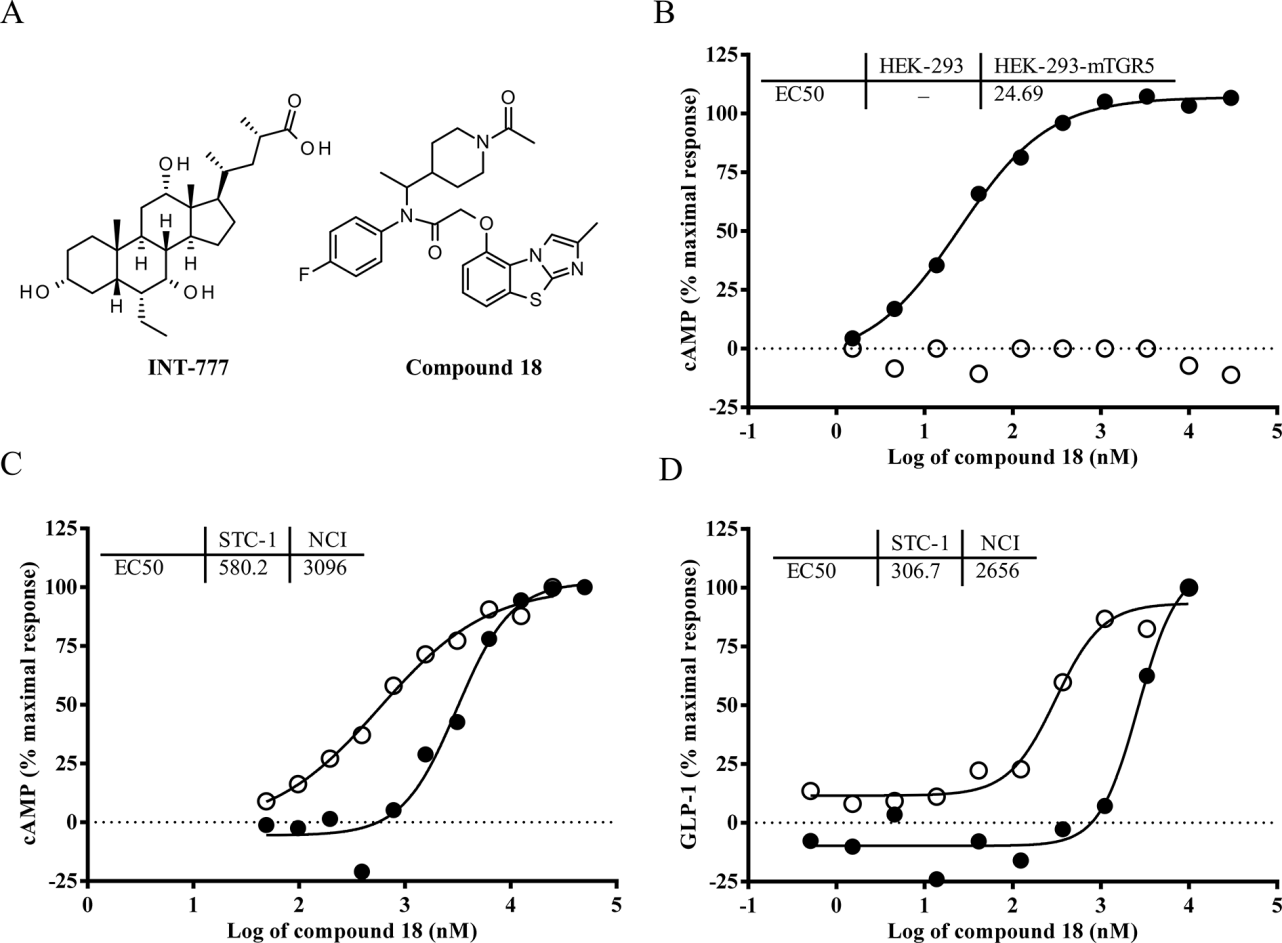
Compound 18 demonstrates potent cAMP production and GLP-1 secretion in mouse cells, but weaker cAMP production and GLP-1 secretion in human cells. The compound structure for both INT-777 and compound 18 are shown (A). HEK-293 cells (white circles) or HEK-293 cells stably transfected with the mouse TGR5 gene (black circles) were treated with compound 18 and assayed for cAMP production (B). Human (NCI-H716, black circles) or mouse (STC-1, white circles) enteroendocrine cells were treated with compound 18 and assayed for cAMP production (C) and GLP-1 secretion (D).

Compound 18 is a novel small molecule agonist of TGR5 developed by Eli Lilly and Company (MW = 508.62, Fig 2A). In order to demonstrate specificity of compound 18 to the TGR5 receptor, we measured compound 18-stimulated cAMP production in HEK-293 cells versus HEK-293 cells stably transfected with the mouse TGR5 gene. In HEK-293 cells, compound 18 showed no elevation of cAMP up to 30000 nM. In HEK-293 cells over-expressing mouse TGR5, compound 18 showed a dose-dependent increase in cAMP with an EC50 of 24.7 nM (Fig 2B **)**. In order to determine potency of compound 18 for the mouse and human TGR5 receptor in cells with endogenous levels of receptor, we measured compound 18-stimulated cAMP production and GLP-1 secretion in both mouse (STC-1) and human (NCI-H716) enteroendocrine cell lines, that we have shown to express TGR5 natively (Fig 1). In STC-1 cells, compound 18 demonstrated robust cAMP production (EC_50_ = 580 nM) and GLP-1 secretion (EC_50_ = 307 nM), while in NCI-H716 cells, compound 18 demonstrated less potent effects on cAMP production (EC_50_ = 3096 nM) and GLP-1 secretion (EC_50_ = 2656 nM) (Fig 2C and 2D). Taken together, these results demonstrate that compound 18 is more than 5-fold more potent on the mouse versus the human TGR5 receptor. In the same assay, INT-777 showed very low potency to promote cAMP production in STC-1 cells (EC_50_ = > 100 μM), and due to its weak activity in the cAMP assay, it was not tested on GLP-1 secretion. Effects of INT-777 in NCI-H716 cells were not investigated, but have been reported previously by others [22].

In addition, no activity has been observed for compound 18 on any target other than TGR5; compound 18 was shown to be inactive against a broad panel of enzymes, receptors, and ion channels (EC50s >125 μM for the enteroendocrine GPCRs GPR120, GPR40, and GPR119, as well as nuclear hormone receptor FXR; IC50s > 10 μM for 56 assays in a CEREP mini panel). Overall, these results show that compound 18 is a highly selective and potent agonist to mouse TGR5 that is more efficacious than INT-777 *in vitro*.

### Compound 18 stimulates GLP-1 and PYY secretion in C57Bl/6 mice

**Fig 3:**
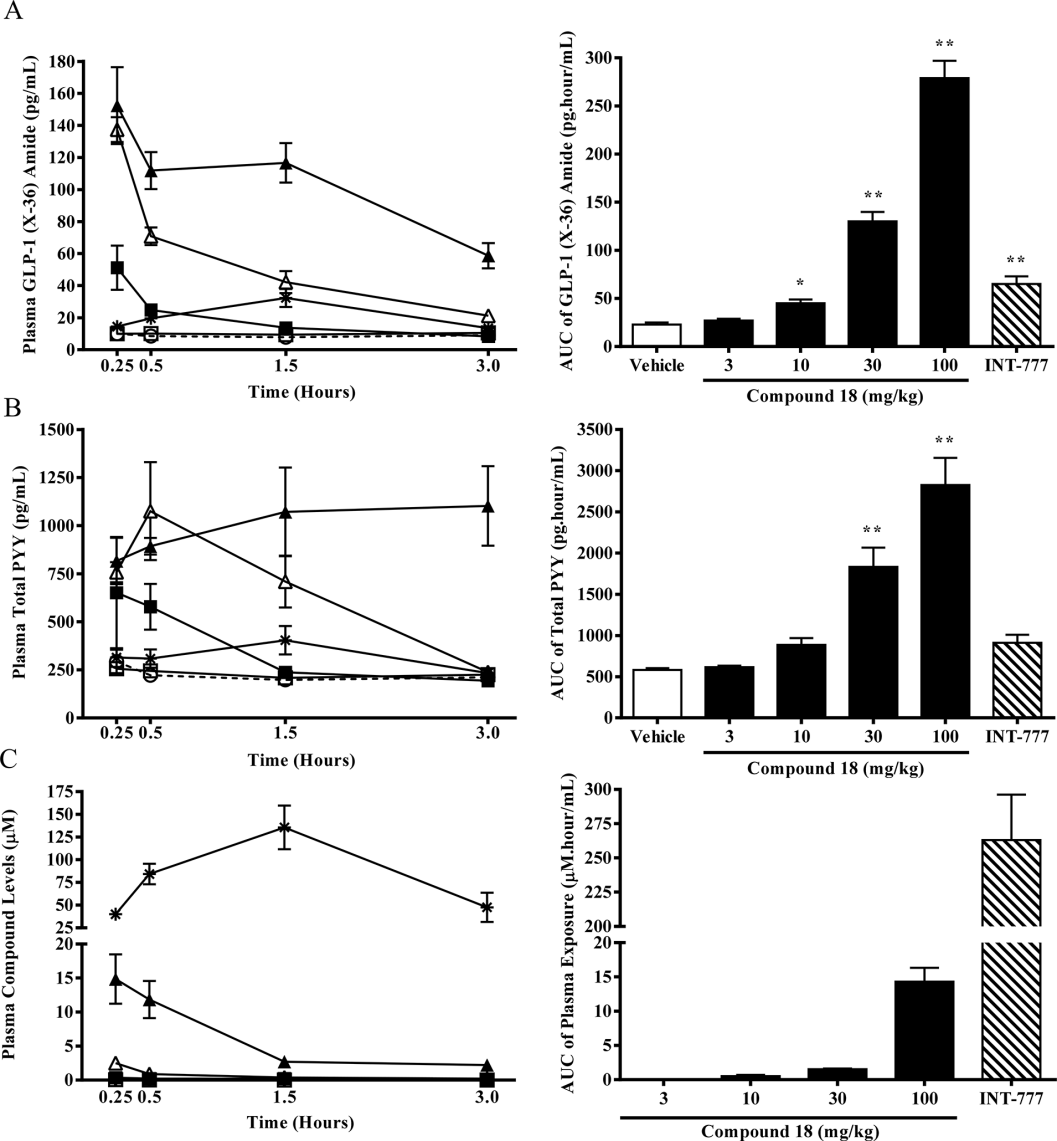
Compound 18 stimulates GLP-1 and PYY secretion in C57Bl/6 mice. Overnight-fasted C57BL/6 mice were orally dosed with vehicle (white circles), compound 18 at 3 mg/kg (white squares), 10 mg/kg (black squares), 30 mg/kg (white triangles), or 100 mg/kg (black triangles), or INT-777 at 100 mg/kg (stars), followed 0.25, 0.50, 1.50, or 3.00 hours post dose by cardiac puncture. Plasma was analyzed for GLP-1 (X-36) amide (A), total PYY (B), or compound levels of INT-777 or compound 18 (C). Resulting raw data was used to generate area under the curve (AUCs) analyses. *n* = 5 per time-point; mean +/- SEM. * *P < 0*.*05*. ** *P < 0*.*01*.

Due to the potent cAMP production and robust GLP-1 secretion observed in the STC-1 cells with compound 18, we hypothesized that oral administration of compound 18 to mice would induce GLP-1 and PYY secretion *in vivo*. To test this, we orally challenged overnight-fasted C57BL/6 mice with 100 mg/kg of INT-777 or 3, 10, 30, or 100 mg/kg of compound 18. Blood was collected at various times post-dose, GLP-1 and PYY were analyzed, and the AUC for GLP-1 and PYY concentrations in plasma over a 3-hour time course was calculated. INT-777 induced a small but significant increase in the AUC of GLP-1 (2.8-fold over vehicle) (Fig 3A), but a non-significant increase in the AUC of PYY (1.6-fold over vehicle) (Fig 3B). Conversely, compound 18 induced robust secretion of GLP-1 (AUC increased 1.2, 2.0, 5.7, and 12.1-fold over vehicle for 3, 10, 30, and 100 mg/kg, respectively) (Fig 3A), as well as elevation in PYY secretion (AUC increased 1.1, 1.5, 3.1, and 4.9-fold over vehicle for 3, 10, 30, and 100 mg/kg, respectively) (Fig 3B). Lastly, plasma compound levels for compound 18 were relatively low [T_max_ = 15 min, C_max_ = 0.059, 0.348, 2.511, and 14.844 μM), AUC_0–3 hr_ = 0.049, 0.507, 1.516, and 14.313 μM·hr for 3, 10, 30, and 100 mg/kg, respectively] compared to the high exposure of INT-777 dosed at 100 mg/kg [T_max_ = 90 min, C_max_ = 135.699 μM, AUC_0–3 hr_ = 264.063 μM·hr] (Fig 3C). Together, these results demonstrate that compound 18 is superior to INT-777 on stimulating GLP-1 and PYY secretion in mice.

### Compound 18 improves glucose tolerance in OGTT experiments in C57Bl/6 mice

**Fig 4:**
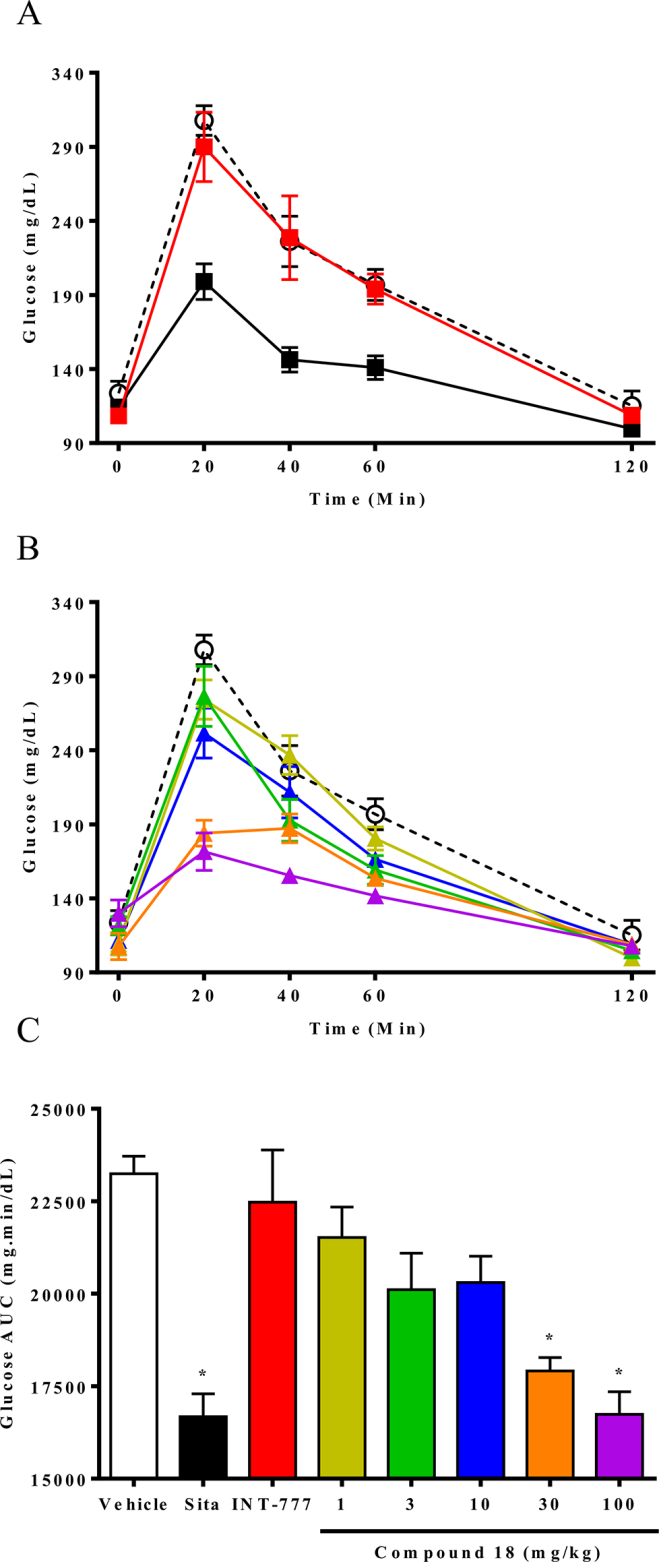
Compound 18 improves glucose tolerance in an OGTT in C57Bl/6 mice. Overnight-fasted C57BL/6 mice were orally dosed with vehicle (white circles), sitagliptin (sita) at 10 mg/kg (black squares), or INT-777 at 100 mg/kg (red squares) (A), or with vehicle (white circles), compound 18 at 1 mg/kg (yellow triangles), 3 mg/kg (green triangles), 10 mg/kg (blue triangles), 30 mg/kg (orange triangles), or 100 mg/kg (purple triangles) (B), followed 15 minutes later with glucose (3 g/kg). Glucometers were used to measure glucose at 0, 20, 40, 60, and 120 minutes post glucose (A and B), and the resulting raw data used to generate area under the curve analyses (AUCs) (C). *n* = 5; mean +/- SEM. * *P < 0*.*01*.

Due to the strong GLP-1 and PYY stimulation of compound 18 in C57BL/6 mice, we hypothesized that compound 18 would improve glucose tolerance. To test this, we performed OGTTs in C57BL/6 mice treated orally with either a dose-range of compound 18, a single dose (100 mg/kg) of INT-777, or a single dose (10 mg/kg) of the DPP4 inhibitor sitagliptin (a dose previously shown to result in maximal DPP4 inhibition; data not shown) as a positive control. An oral challenge of glucose (3 g/kg) was given 15 min after compound administration since compound 18-induced peaks in GLP-1 were observed at this time-point. INT-777 had no detectable improvement of glucose tolerance despite substantially higher plasma exposure (Fig 4A and 4C), while compound 18 reduced glucose excursions in mice at doses equal to and greater than 30 mg/kg (Fig 4B and 4C).

**Fig 5:**
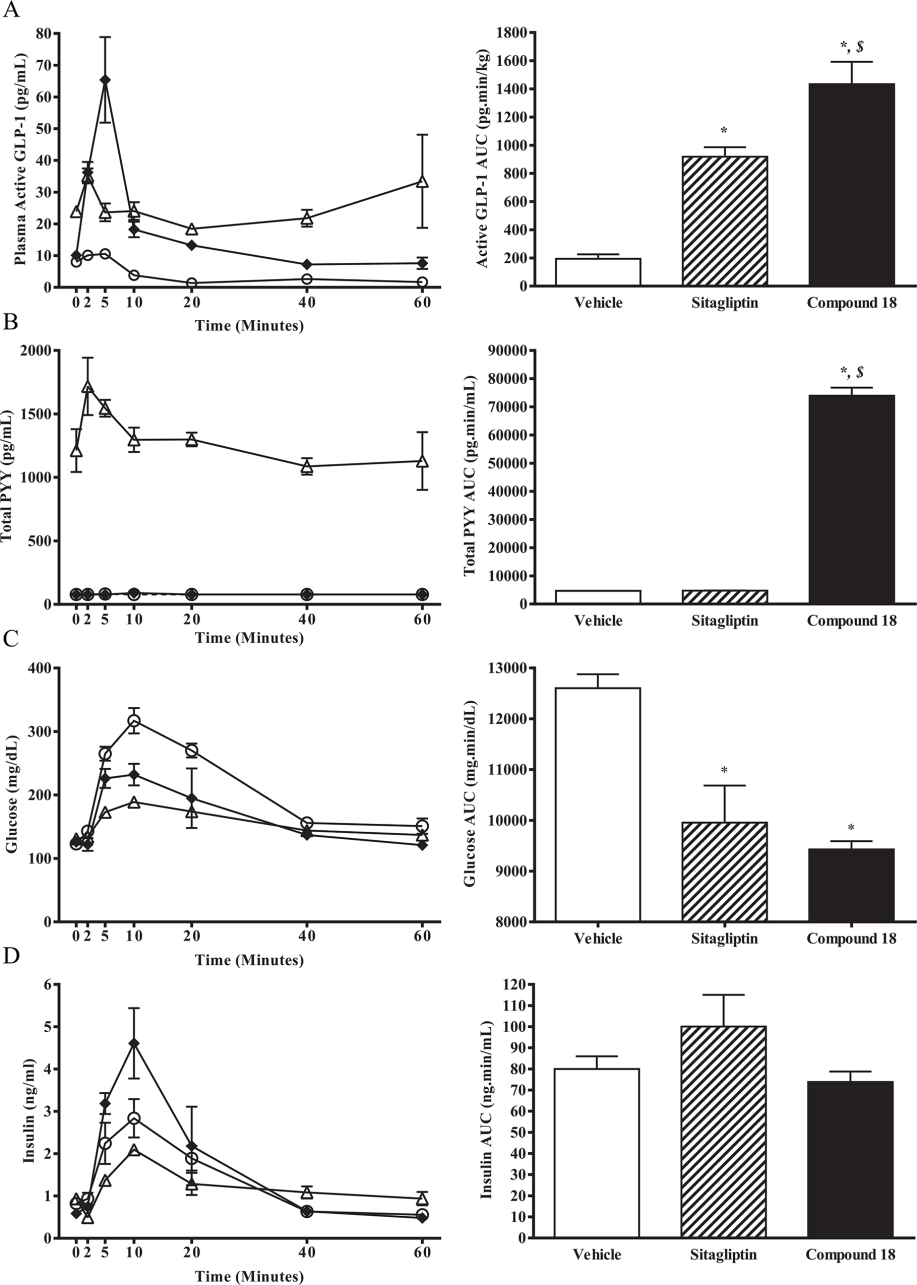
Compound 18 increases GLP-1 and PYY and lowers the glucose and insulin excursions as compared to sitagliptin in an OGTT in C57BL/6 mice. Overnight-fasted C57BL/6 mice were orally dosed with vehicle (white circles), compound 18 at 30 mg/kg (white triangles), or sitagliptin at 10 mg/kg (black diamonds), followed 15 minutes later by an oral dose of glucose (3 g/kg). Plasma was analyzed for active GLP-1 (A), total PYY (B), and insulin (D), while blood glucose was measured by glucometers (C). Resulting raw data was used to generate area under the curve (AUC) analyses. *n* = 5 per time-point; mean +/- SEM. * *P < 0*.*05* versus vehicle. *$ P < 0*.*05* versus sitagliptin.

While compound 18 showed robust GLP-1 and PYY stimulation in fasted mice (Fig 3A and 3B), and lowered glucose excursions in an OGTT (Fig 4), it remained unclear how compound 18 affected GLP-1, PYY, and insulin in the context of the OGTT experiment. To measure hormone levels along with glucose concentrations, overnight-fasted C57BL/6 mice were orally challenged with glucose (3 g/kg) 15 minute after administration of 30 mg/kg of compound 18 or a single oral dose of sitagliptin (10 mg/kg). To evaluate glucose, insulin, GLP-1, and PYY levels throughout the OGTT, each time-point consisted of a separate group of mice that were used to record glucose by use of a glucometer followed by terminal bleeds for measurement of active GLP-1, total PYY, and insulin. AUCs were then calculated for the entire hour of the OGTT. Our results demonstrate that compound 18 induced a prolonged increase in active (intact) GLP-1 resulting in a superior AUC for active GLP-1 above both vehicle and sitagliptin (Fig 5A), as well as a higher increase in the AUC of total PYY above both vehicle and sitagliptin (Fig 5B), that likely result in a lowering of the glucose excursion by compound 18 to levels indistinguishable from those of sitagliptin-treated animals (Fig 5C). Changes in insulin AUCs were not increased with treatment of either sitagliptin or compound 18, likely due to the lower glucose levels reducing the drive for glucose-stimulated insulin secretion (Fig 5D).

### Chronic administration of compound 18 to mice does not cause TGR5 desensitization and leads to weight loss

**Fig 6:**
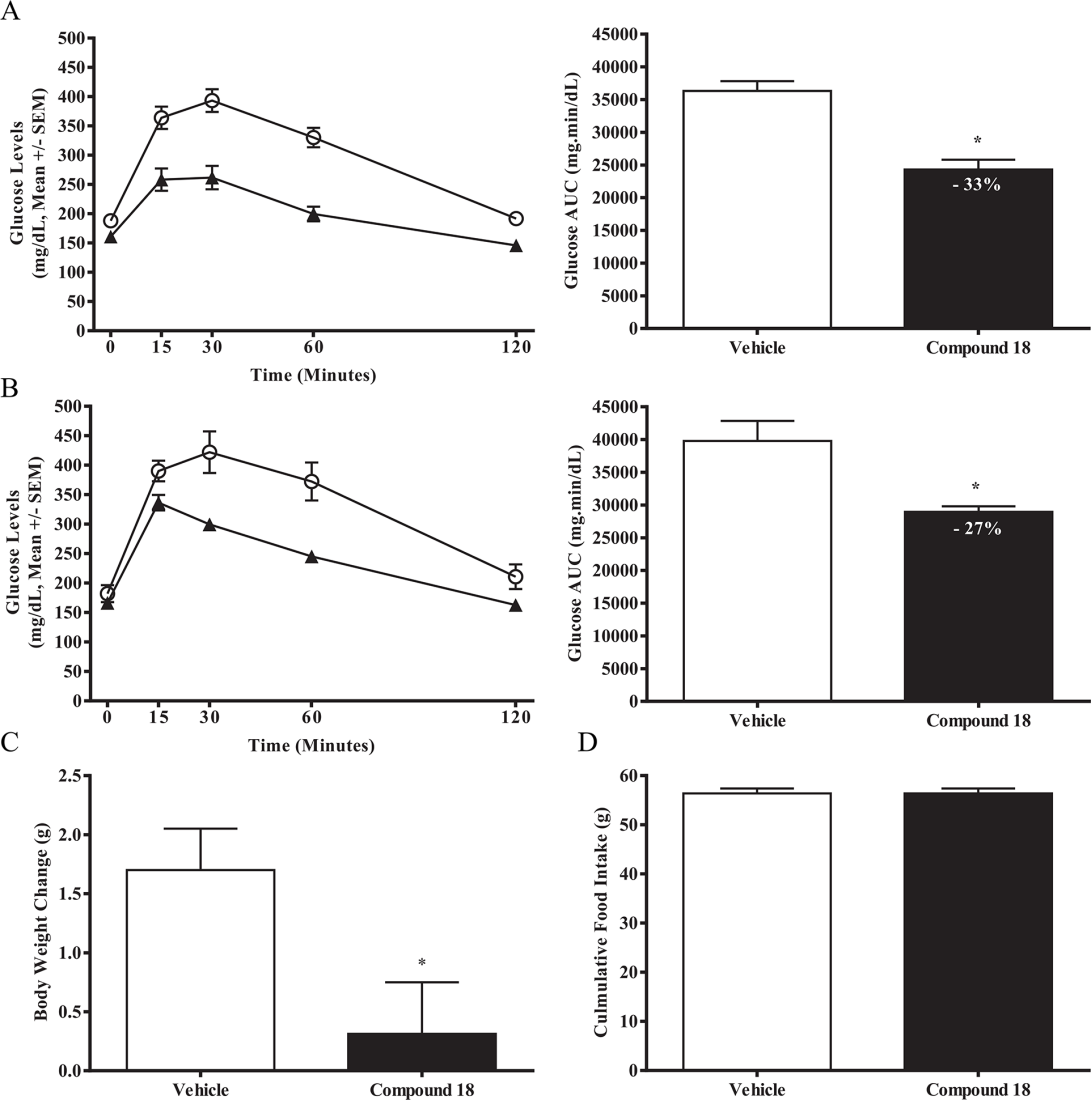
Chronic administration of compound 18 to mice does not cause desensitization and also leads to weight loss. Diet-induced obese (DIO) C57BL/6 mice were either dosed acutely or chronically for two weeks with vehicle (white circles, white bars) or compound 18 at 60 mg/kg (black triangles, black bars). An OGTT on over-night fasted DIO mice was performed after either a single dose (A, acute) or after two weeks of dosing (B, chronic), using 2 g/kg glucose dosed 30 minutes after compound dosing. Changes in body weights (C) and cumulative food intake (D) were recorded for the two week period. *n* = 8; mean +/- SEM. * *P < 0*.*01*.

While compound 18 led to significant glucose lowering in an OGTT studies when dosed acutely, it remained unclear if the mechanism of TGR5-stimulated GLP-1 secretion desensitizes over time. In addition, oral dosing of both bile acid (cholic acid) and INT-777 has been previously reported to result in weight loss in diet-induced obese (DIO) mice with chronic administration [20,22]. In order to determine if desensitization occurs, as well as explore the potential for changes in body weight by compound 18, we obtained DIO C57BL/6 mice and randomly assigned them into either an acute or chronic study. For the acute experiment, overnight-fasted DIO mice were dosed with vehicle or 60 mg/kg of compound 18 the next morning, and an OGTT was performed 30 minutes after compound dosing using 2 g/kg glucose. For the chronic study, DIO mice were orally dosed with vehicle or 60 mg/kg compound 18 once a day for two weeks; body weights and food intake were measured during the treatment period. After two weeks, mice were over-night fasted, dosed the following morning, and subjected to an OGTT 30 minutes after compound dosing. A similar reduction in the glucose excursion was shown in the acute administration of compound 18 (Fig 6A, 33 ± 5 percent reduction) as compared with chronic administration of compound 18 (Fig 6B, 27 ± 6 percent reduction), indicating that TGR5 agonism by this compound does not desensitize over time. Moreover, the chronically-dosed DIO mice demonstrated a small, but significant reduction of weight gain (1.4 g reduction in body weight gain) (Fig 6C) in compound 18-treated mice versus vehicle over the two week period, with no change in food intake (Fig 6D). Compound 18 treated mice showed a trend towards reduced gain in fat mass (p = 0.067) with no difference in lean mass (P = 0.995) (data not shown), suggestive that the effects on body weight gain are likely mediated by changes in body fat.

### Compound 18 causes TGR5-dependent gallbladder filling at doses that do not induce GLP-1 secretion

**Fig 7:**
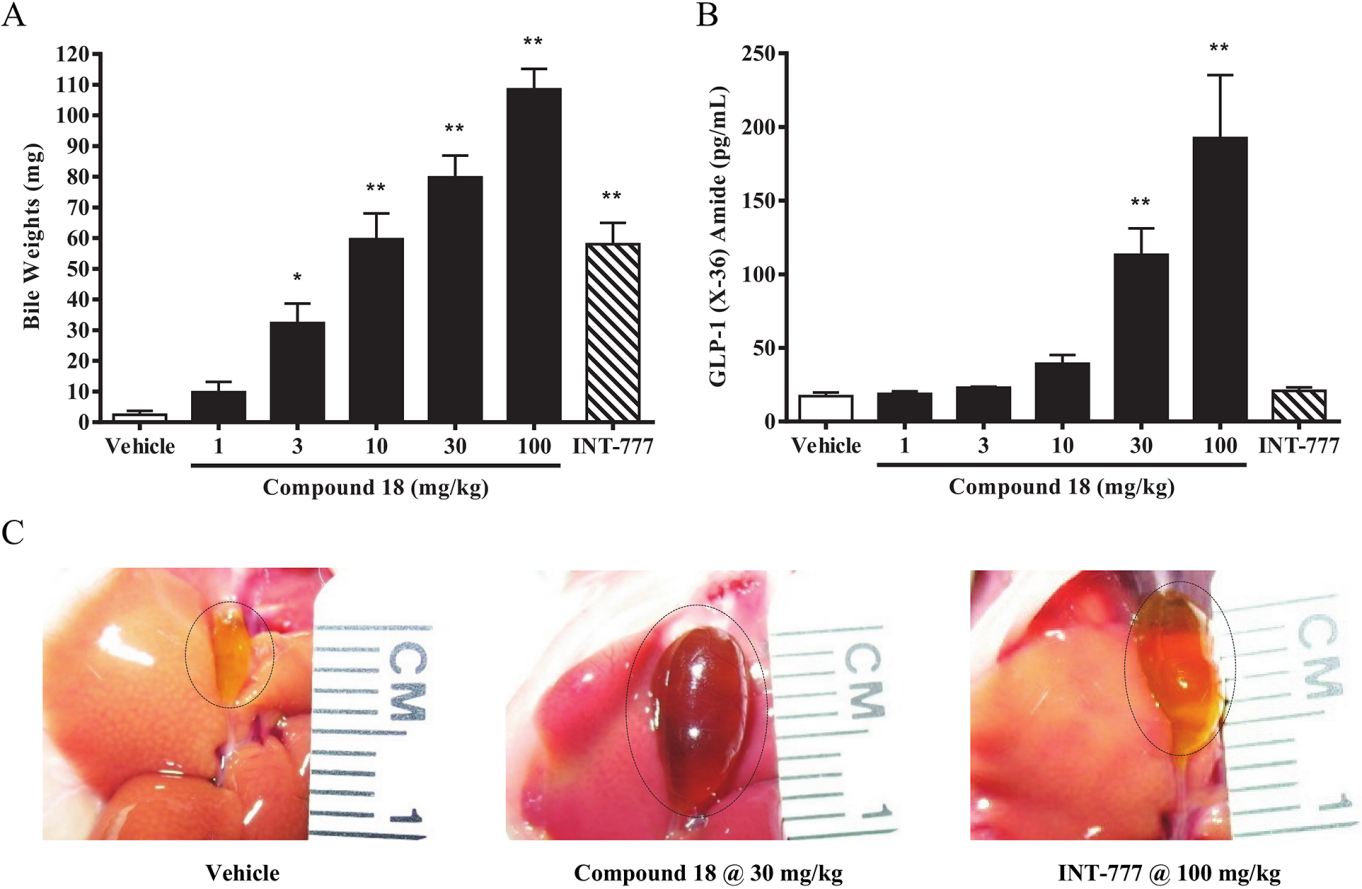
Compound 18 causes gallbladder filling at doses that do not induce GLP-1 secretion. C57BL/6 mice were orally dosed with vehicle, compound 18 at 1, 3, 10, 30, or 100 mg/kg, or INT-777 at 100 mg/kg, for 3 days twice a day (BID), with over-night fasting performed on the last evening. On the third day, compound was dosed in the morning followed 30 minutes later by measurement of bile filling (A) by use of a needle and syringe and GLP-1 (x-36) amide (B) by cardiac puncture. Representative photos of gallbladder filling in animals treated 3 days with vehicle, compound 18 at 30 mg/kg, and INT-777 at 100 mg/kg; gallbladders highlighted with dotted circles (C). *n* = 5; mean +/- SEM. * *P < 0*.*05*. ** *P < 0*.*01*

In addition to expression in intestinal tissues, TGR5 is also expressed at high levels in gallbladder (Fig 1). Importantly, it has been shown that administration of the natural bile acid lithocholic acid or the synthetic bile acid analog INT-777 stimulate gallbladder filling with bile in *wild-type* but not *Tgr5* KO mice, suggestive of a TGR5-dependent mechanism [24]. While these data suggested that TGR5 agonists pose a risk for gallbladder-related conditions, the studies were limited to bile acid and bile acid analogs. To test this phenomenon using a non-bile acid small molecule agonist of TGR5, we orally dosed C57BL/6 mice twice per day (BID) for three days with compound 18, with overnight fasting performed on the last evening, followed by assessment of gallbladder filling by measuring bile weight, as well as measuring subsequent GLP-1 levels in blood 30 minutes after the last dose. Results demonstrated a clear dose dependent increase in bile weight (Fig 7A) as well as elevated GLP-1 levels (Fig 7B) with compound 18, while INT-777 at 100 mg/kg induced a significant increase in bile weight (Fig 7A) but no detectable increase in GLP-1 (Fig 7B). The changes in plasma GLP-1 were not associated with any changes in gene expression of the *Gcg* gene (the transcript that encodes GLP-1) or *Gpbar1* (the gene encoding TGR5) in ileum suggesting that the TGR5 agonist effect is primarily due to stimulation of GLP-1 secretion rather than altering gene expression (data not shown). To better comprehend the magnitude of the increase in gallbladder filling as measured by bile weight with compound 18 and INT-777, representative photos were taken of gallbladders of mice treated for three days with either vehicle, compound 18 at 30 mg/kg, or INT-777 at 100 mg/kg (Fig 7C). Importantly, significant effects on bile weight were observed with compound 18 at doses as low as 3 mg/kg, while significant effects on GLP-1 secretion with compound 18 were limited to doses of 30 mg/kg and above. Moreover, effects on lowering of the glucose excursions in an OGTT were limited to doses of compound 18 at 30 mg/kg and higher (Fig 4). Together, these data suggest that TGR5 agonism in mice has an increased potential to induce gallbladder filling versus inducing either GLP-1 secretion or lowering glucose excursions in an OGTT.

**Fig 8:**
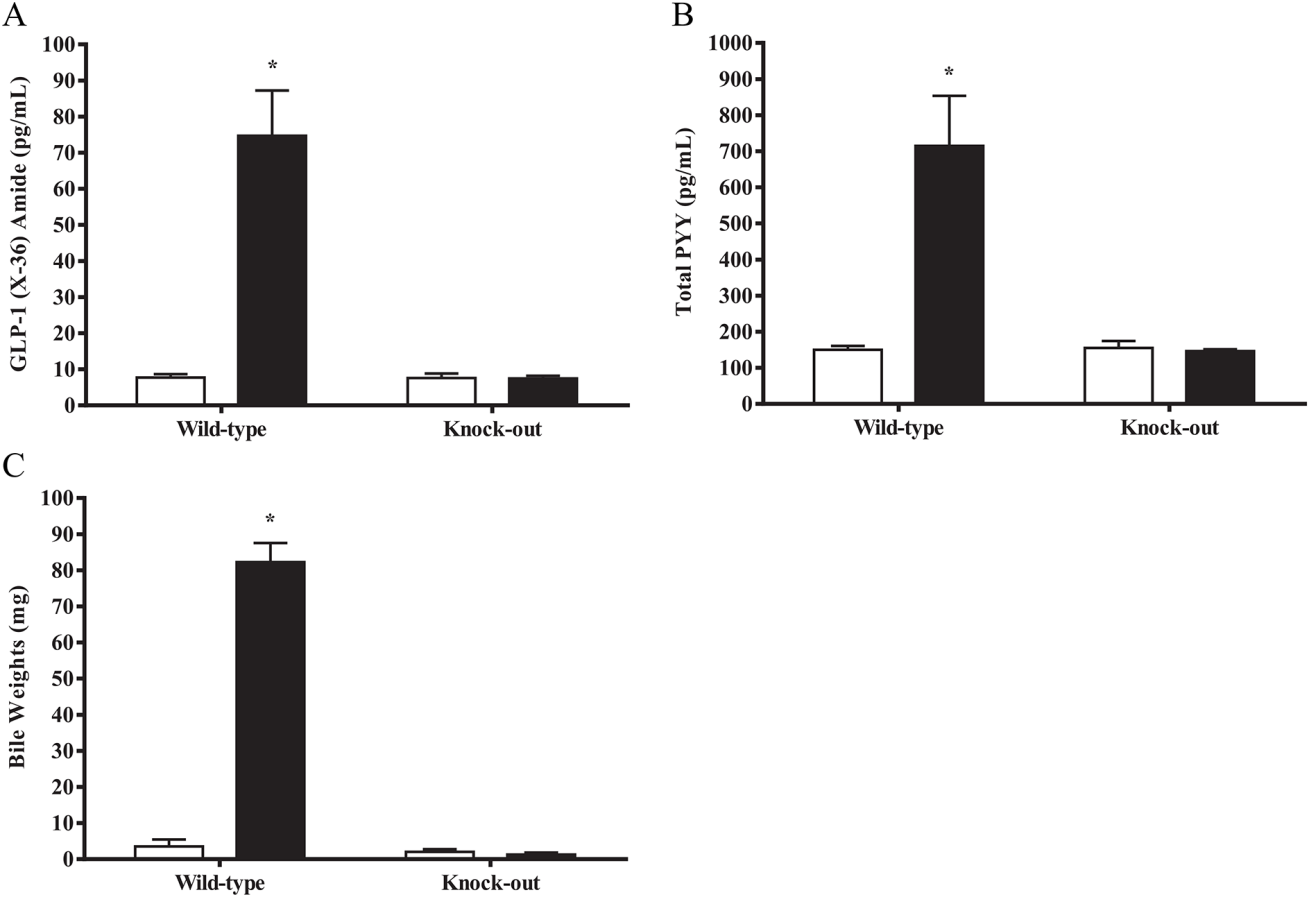
Compound 18 fails to show effects on GLP-1, PYY, and gallbladder filling in TGR5 knockout mice. TGR5 knock-out and wild-type mice were orally dosed with vehicle (white bars) or compound 18 at 100 mg/kg (black bars) for 3 days twice a day (BID), with over-night fasting performed on the last evening. On the third day, compound was dosed in the morning followed 30 minutes later by measurement of GLP-1 (x-36) amide (A) and total PYY (B) by cardiac puncture and bile weight (C) by use of a needle and syringe. *n* = 5; mean +/- SEM. * *P < 0*.*01*.

To demonstrate that the effects on GLP-1, PYY, and gallbladder filling is a TGR5-dependent mechanism induced by compound 18, we treated *Tgr5* KO and *wild-type* littermates with 100 mg/kg of compound 18 for 3 days BID, followed by measurement of GLP-1, PYY, and bile weight at 30 minutes after the last dose. These experiments demonstrate a significant increase in GLP-1 (Fig 8A), PYY (Fig 8B), and bile weight (Fig 8C) in *wild-type* mice treated with compound 18, but no detectable increase in GLP-1, PYY, or bile weight in *Tgr5* KO mice. Overall, these results demonstrate that compound 18 induces effects on gut hormones (GLP-1 and PYY) and gallbladder filling (bile weight) in a TGR5-dependent manner.

## Discussion

Incretin-based therapies, notably DPP4 inhibitors and GLP-1 analogs, have shown great promise in the treatment of type 2 diabetes. Similarly, a TGR5 agonist that is capable of inducing the secretion of GLP-1 as well as increasing energy expenditure may prove to be a promising incretin-based strategy for the treatment of type 2 diabetes and obesity [26,27]. To date, the list of reported TGR5 agonists is small. The bile acid derivative INT-777 increases cAMP and GLP-1 secretion *in vitro* and possesses effects on acute GLP-1 secretion *in vivo* [22]. Here, our results show that INT-777 has only a minor effect on cAMP production *in vitro* and displays only a small increase in GLP-1 secretion and no detectable increase in PYY *in vivo*. Moreover, the effect on GLP-1 from acute administration of INT-777 is insufficient to reduce the glucose excursion in an OGTT. While chronic treatment of obese mice with INT-777 has been shown to improve the glucose excursion in an OGTT [22], this effect on glucose is likely due to weight loss observed in these mice and not due to direct effects of TGR5 agonism on GLP-1 and indirect effects on insulin secretion. In addition to bile acids and bile acid analogs, several small molecule agonists of TGR5 have been reported to stimulate plasma GLP-1 secretion in vivo and lower glucose excursions in an OGTT, including compound 23g, TRC210258, compound 22e, compound 45h, compound 9r, and compound 19 [28–31]. Nevertheless, all TGR5 compounds tested on gallbladder (compound 23g, compound 9r, and compound 19) showed gallbladder filling at the same dose of compound that lowered glucose in the OGTT [28,31]. Overall, recent evidence suggests that small molecule agonists of TGR5 carry the same risk of gallbladder filling in mice as seen originally with bile acid and bile acid analogs. Nevertheless, it has yet to be effectively demonstrated that gallbladder filling cannot be separated on a dose/exposure basis from increases in GLP-1 and resulting lowering of glucose excursions in an OGTT. Moreover, it has yet to be demonstrated that these effects on GLP-1, and especially the effects on gallbladder filling via these agonists, is TGR5 mediated. Importantly, our investigation of compound 18 showed pronounced cAMP production and GLP-1 secretion, and a robust effect on acutely inducing GLP-1 secretion in mice that, in turn, led to reduced glucose excursion in an OGTT in mice. Interestingly, compound 18 resulted in similar reductions in glucose excursions in an OGTT in DIO mice when dosed either acutely or after 14 days of dosing that cannot be attributed to modestly reduced weight gain over this two week period. Overall, these results demonstrate that compound 18 is superior to INT-777 on inducing GLP-1 as well as lowering glucose in mice in a weight-independent manner.

TGR5 has been previously shown to have 10-fold higher expression in mouse and human gallbladder than any other tissue [15,23], which was confirmed by our findings. Furthermore, TGR5 is expressed in the gallbladder epithelium and has a role in fluid secretion [23]. Together, these suggest that TGR5 agonists may affect gallbladder physiology. In fact, gallbladder filling with bile was observed in *wild-type* but not *Tgr5* KO mice treated either with 0.2% cholic acid in the diet for two weeks or with acute administration of lithocholic acid or INT-777 [24]. Moreover, compound 23g, compound 9r, and compound 19a increased gallbladder filling at the same 50 mg/kg dose as produced positive effects on GLP-1 secretion and glucose lowering in OGTT studies [28,31]. These data suggest a potential of TGR5-induced effects on gallbladder physiology.

Compound 18 is a potent and specific non-bile acid small molecule agonist of TGR5 that shows dose-dependent effect on gallbladder filling, a phenomenon completely absent in *Tgr5* KO mice. Moreover, effects on GLP-1 secretion and glucose lowering did not occur at doses where significant elevations in gallbladder filling occur. In addition, in our studies, INT-777 showed little to no elevations in GLP-1 secretion but did demonstrate a significant elevation in gallbladder filling. Together, these data suggest gallbladder filling is a TGR5 dependent effect, and a phenomenon that is more sensitive to TGR5 agonism than GLP-1 secretion and effects on glucose homeostasis, a finding that is in line with the expression profile of TGR5.

One potential way to avoid TGR5 agonist–induced gallbladder filling is the development of gut-restricted TGR5 agonists, allowing stimulation of the intestinal L-cells to produce GLP-1 and PYY, while reducing or eliminating exposure of an agonist to the gallbladder. Unfortunately, while compound 18 had low systemic exposure [C_max_ = 0.059 μM, AUC_0–3 hr_ = 0.049 μM·hr for 3 mg/kg], it was still sufficient to cause gallbladder filling at this dose / exposure. A true gut-restricted and efficacious TGR5 agonist has yet to be described. Overall, these results using a highly selective and potent small molecule agonist of TGR5 demonstrate the magnitude of TGR5 agonists to stimulate GLP-1 and PYY secretion *in vivo*, the likelihood of TGR5 agonists to lead to weight loss in chronic studies, and the potential of TGR5 agonists to lower glucose. Unfortunately, these results also demonstrate the physiological consequences of TGR5 agonism at the gallbladder, confirming the studies performed by Li *et al*. [24]. Importantly, our results further demonstrate that gallbladder filling is a prominent phenotype of TGR5 agonism that cannot be separated from elevations in GLP-1 or subsequent effects on glucose homeostasis, even with TGR5 agonists possessing low systemic exposure. Overall, these data demonstrate the potential benefits and risks, of using TGR5 agonists to treat diabetes and metabolic diseases.
